# Rifaximin Versus Norfloxacin for Secondary Prophylaxis of Spontaneous Bacterial Peritonitis in the Era of Antimicrobial Resistance: A Systematic Review and Meta-Analysis with Missing-Data Sensitivity Analyses

**DOI:** 10.3390/biomedicines14102285

**Published:** 2026-10-09

**Authors:** Jing-Hong Hu, Ming-Ling Chang, Nai-Jen Liu, Kai-Feng Sung, Yung-Yu Hsieh, Chun-Hsien Chen

**Affiliations:** 1 Division of Gastroenterology and Hepatology, Department of Internal Medicine, Yunlin Chang Gung Memorial Hospital, Yunlin County 638, Taiwan; 2 Department of Gastroenterology and Hepatology, Linkou Chang Gung Memorial Hospital, Taoyuan 333, Taiwan; 3 College of Medicine, Chang Gung University, Taoyuan 333, Taiwan; 4 Division of Gastroenterology and Hepatology, Department of Internal Medicine, Chiayi Chang Gung Memorial Hospital, Chiayi County 613, Taiwan

**Keywords:** spontaneous bacterial peritonitis, cirrhosis, rifaximin, norfloxacin, secondary prophylaxis, antimicrobial resistance, meta-analysis

## Abstract

**Background**: Fluoroquinolone secondary prophylaxis of spontaneous bacterial peritonitis (SBP) was established in a fluoroquinolone-naïve era. We re-appraised the randomized evidence comparing rifaximin with norfloxacin and its robustness to missing outcome data and competing mortality. **Methods**: We searched MEDLINE, Embase, CENTRAL, and Scopus through 15 July 2026. The primary estimand was the intention-to-treat risk ratio (RR) for six-month SBP recurrence, with death as a competing event. Trials were pooled with REML random-effects models and Hartung–Knapp–Sidik–Jonkman (HKSJ) intervals; missing-outcome bounding across all trials and a composite of recurrence or death were post hoc. Certainty was rated with GRADE. The review was registered retrospectively in PROSPERO (CRD420261501120). **Results**: In three randomized trials (398 randomized patients; none double-blind), rifaximin was associated with fewer recurrences than norfloxacin (RR 0.21, 95% HKSJ CI 0.05–0.89), but the modified HKSJ interval crossed unity (0.03–1.31). Across plausible missing-outcome scenarios, the Mantel–Haenszel RR ranged from 0.15 to 0.73 (worst case 95% CI 0.45–1.17) and for recurrence or death from 0.35 to 0.75 (0.55–1.03). Certainty was very low. **Conclusions**: Rifaximin may reduce SBP recurrence compared with norfloxacin, but the evidence is very uncertain and sensitive to unreported outcomes; a contemporary, blinded trial is needed before guideline change.

## 1. Introduction

Spontaneous bacterial peritonitis (SBP) is a severe, potentially life-threatening infection of ascitic fluid that arises without a surgically treatable intra-abdominal source and represents one of the defining complications of decompensated cirrhosis [1]. Even after a first episode is successfully treated, the cumulative probability of recurrence within one year approaches 70% [2], and each recurrence carries substantial mortality and accelerates the trajectory toward acute-on-chronic liver failure [3,4]. On the strength of this recurrence risk, international guidelines—including those of the American Association for the Study of Liver Diseases (AASLD) and the European Association for the Study of the Liver (EASL)—have for three decades recommended indefinite oral fluoroquinolone secondary prophylaxis, most commonly norfloxacin or ciprofloxacin, as standard care [5,6]. That recommendation rests largely on a small number of trials conducted in the late twentieth century, when fluoroquinolone-naïve gut flora made these agents both highly active and durably protective.

The microbiological premise underlying those trials has since weakened. Decades of population-wide fluoroquinolone exposure have driven a marked rise in infections caused by multidrug-resistant (MDR) organisms, extended-spectrum β-lactamase-producing Enterobacterales, and fluoroquinolone-resistant Gram-negative bacteria [3,4]. In cirrhosis specifically, this shift compromises the regimens intended to prevent SBP, raises the likelihood that empirical therapy for breakthrough infection will fail, and is associated with higher mortality [7]. Whether long-term fluoroquinolone prophylaxis remains net-beneficial in the contemporary resistance landscape has therefore become an open question [8].

Rifaximin, a poorly absorbed, gut-selective rifamycin used chiefly for hepatic encephalopathy, achieves high intraluminal concentrations with negligible systemic exposure [9,10] and has been reported to exert eubiotic effects on the gut microbiome and intestinal barrier [11]. These properties have made it a candidate alternative to systemic fluoroquinolones, and it has been examined in several head-to-head trials.

The comparative evidence has already been synthesized in part. A Cochrane network meta-analysis of 29 trials, a Bayesian network meta-analysis by Soni et al., and a pairwise review by Song et al. all included rifaximin–norfloxacin comparisons, and the latter two reported estimates for secondary prophylaxis [12,13,14]; an earlier pairwise review addressed rifaximin for SBP prophylaxis generally [15]. These reviews, however, pooled available-case data without examining the analysis populations of the contributing trials, did not incorporate the large observational studies of secondary prophylaxis published in 2025–2026, and, in the case of the network meta-analyses, rated the secondary-prophylaxis evidence as of low or very low certainty without specifying the sources of that uncertainty.

The present review therefore does not claim to be the first synthesis of this comparison. Its purpose is a focused re-appraisal: to pool the randomized head-to-head evidence for secondary prophylaxis with small-sample methods appropriate to three trials, to test the robustness of that estimate to the missing outcome data that we identified in the largest trial, and to place the result alongside contemporary cohort evidence on the declining effectiveness of fluoroquinolone-based secondary prophylaxis.

## 2. Methods

### 2.1. Design, Reporting, and Registration

This systematic review and meta-analysis is reported according to PRISMA 2020 [16] (completed checklist in Supplementary Table S1). The review was registered retrospectively in PROSPERO (CRD420261501120) on 8 September 2026, after the literature search (15 July 2026) and after completion of study selection, data extraction, and the initial analyses (1 September 2026); registration therefore does not document pre-specification of any analysis. Quantitative pooling was restricted to the rifaximin–norfloxacin comparison because it was the only drug pair evaluated in more than one randomized comparison; this decision was made on 7 August 2026, after study selection, and is data-informed. The modified HKSJ interval, the missing-outcome scenarios and the generalized linear model were specified after data extraction; the intention-to-treat estimand with death as a competing event, the extension of the missing-outcome scenarios to all trials, the composite outcome of recurrence or death, and the log-link generalized linear model were adopted during peer review. All of these analyses are labeled post hoc throughout.

### 2.2. Search Strategy

We searched MEDLINE (via PubMed), Embase, the Cochrane Central Register of Controlled Trials (CENTRAL), and Scopus from inception through 15 July 2026. The database search strategies and hit counts are reported in Supplementary Table S2. No language or date limits were applied. Reference lists of included studies and prior systematic reviews [12,13,14,15] were screened by hand. Full-text exclusions and reasons are reported in Supplementary Table S3.

### 2.3. Eligibility Criteria

We included (1) adults with cirrhosis and at least one prior episode of SBP; (2) head-to-head comparisons of antibiotic regimens for secondary SBP prophylaxis or comparisons of prophylaxis with no prophylaxis or placebo; (3) reporting SBP recurrence, all-cause mortality, or resistant infection; and (4) randomized controlled trials (RCTs) or cohort studies. Studies of primary prophylaxis only, studies in which secondary-prophylaxis outcomes could not be separated, and non-comparative reports were excluded.

### 2.4. Data Extraction and Analysis Populations

Two reviewers (J.-H.H. and M.-L.C.) independently screened records, extracted data using a standardized form, and assessed risk of bias; disagreements were resolved by discussion, with adjudication by a third reviewer (N.-J.L.) when needed. Extracted items are listed in Supplementary Table S4. Event counts, denominators, and deaths were taken from the full texts, including the participant-flow diagrams. Trial investigators were not contacted for unreported data.

For Elfert et al. [17], SBP recurrence is reported for patients completing six months of treatment (rifaximin 4/103; norfloxacin 13/92), although 131 patients were randomized to each arm and the statistical section describes an intention-to-treat analysis; mortality is reported for the randomized population (18/131 vs. 32/131). According to the participant-flow diagram, the 28 rifaximin and 39 norfloxacin non-completers comprised 18 and 32 deaths, 6 and 4 patients lost to follow-up, and 4 and 3 consent withdrawals; recurrence status is therefore unknown for 10 rifaximin and 7 norfloxacin surviving non-completers, and whether any patient who died had recurred before death is not reported. Praharaj et al. [18] randomized 33 patients to each arm of the pre-specified secondary-prophylaxis stratum and analyzed 26 rifaximin and 33 norfloxacin patients per protocol; the seven excluded rifaximin patients (non-compliance or loss to follow-up) have unknown outcomes, whereas deaths among analyzed patients (7/26 vs. 12/33) are reported in the text. Mostafa et al. [19] randomized unequally (40:30) and withdrew the five norfloxacin patients who developed recurrence (the trial endpoint), so recurrence status is known for all 70 randomized patients; no deaths were reported.

### 2.5. Estimand and Outcomes

The primary estimand was the intention-to-treat risk ratio (RR) for SBP recurrence within six months, rifaximin versus norfloxacin, among all randomized patients with cirrhosis and prior SBP, with death before recurrence treated as a competing event: patients who died without documented recurrence were counted as recurrence-free, as in a cumulative-incidence estimand. Randomized patients with unknown outcomes for reasons other than death (Section 2.4) were assumed recurrence-free in the primary analysis, and this assumption was varied in sensitivity analyses. The analysis populations reported by the trials (324 of 398 randomized patients) were analyzed as a sensitivity analysis. Because treating death as a competing event can make the arm with higher mortality appear more favorable, we also analyzed the composite of SBP recurrence or death. Secondary outcomes were all-cause mortality and infection with fluoroquinolone-resistant or MDR organisms.

### 2.6. Risk of Bias and Certainty

RCTs were assessed with RoB 2 [20] and cohort studies with ROBINS-I [21]. Certainty of evidence for the primary comparison was rated with GRADE [22], starting at high for randomized evidence and downgrading for risk of bias, inconsistency, indirectness, imprecision, and publication bias according to GRADE guidance.

### 2.7. Statistical Analysis

Quantitative pooling was restricted to the rifaximin–norfloxacin comparison, the only drug pair evaluated in more than one randomized comparison (Section 2.1); other comparisons were synthesized narratively.

The primary analysis was a random-effects model with the restricted maximum-likelihood (REML) estimator of τ^2^ and HKSJ confidence intervals, chosen because the number of trials was three [23]. Because the HKSJ interval can be narrower than the Wald interval when observed heterogeneity is below expectation, we also report the modified HKSJ interval of Röver et al., which uses the larger of the HKSJ and Wald standard errors [24]. For the trial with zero events in one arm (Mostafa et al. [19], allocated 40:30), 0.5 was added to each cell of that trial only for the inverse-variance analysis. Because a constant correction of 0.5 can bias the estimate when group sizes are unequal [25], we regard the Mantel–Haenszel (MH) fixed-effect RR, computed without continuity correction, as the preferred estimate for that trial’s contribution. A generalized linear model with fixed study effects and a log link (binomial likelihood, log-risk scale, profile-likelihood confidence interval) was also fitted without continuity correction so that all model-based estimates are on the RR scale. Heterogeneity was quantified with Cochran’s Q and I^2^, recognizing that with three trials I^2^ is imprecise and I^2^ = 0% does not exclude heterogeneity.

Sensitivity analyses comprised: (i) leave-one-out analyses, reported with both HKSJ and MH intervals, noting that HKSJ intervals with one degree of freedom are uninformative; (ii) the analysis populations reported by the trials; (iii) post hoc bounding scenarios for all randomized patients with unknown outcomes in Elfert et al. [17] and Praharaj et al. [18] (Mostafa et al. [19] had no missing recurrence data)—B1, all patients with unknown outcomes recurred; B2, recurrence imputed for rifaximin patients only (worst case for rifaximin); B3, recurrence imputed for norfloxacin patients only (best case); and B4, unknown outcomes recurred at twice the observed arm-specific rate (an informative-missingness assumption [26]), together with a deliberately extreme scenario (B5) in which recurrence was also imputed to rifaximin patients who died; and (iv) the composite of recurrence or death, bounded by the range of values compatible with the published data. In Elfert et al. [17], recurrences were reported among completers and deaths among non-completers, so the two events are disjoint, and only the 17 surviving non-completers are uncertain; in Praharaj et al. [18], the overlap between SBP and death was not reported, so the composite was bounded between complete overlap and no overlap; Mostafa et al. [19] reported no deaths. Influence was assessed with a Baujat plot and Cook’s distance in the trial-reported populations. Funnel plots and small-study-effect tests were not performed because they are uninterpretable with three trials. Analyses used R 4.3.3 and metafor version 4.4-0 [27]; the full code is provided as Supplementary Code.

## 3. Results

### 3.1. Study Selection and Characteristics

Of 456 records identified (446 from database searches and 10 from citation searching), 17 reports were assessed for eligibility, and seven studies met the inclusion criteria (Supplementary Figure S1, PRISMA flow; Table 1): four RCTs [17,19,28,29], one pre-specified RCT subgroup [18], and two national cohort studies [30,31]. Three trials compared rifaximin with norfloxacin [17,18,19]; one compared norfloxacin with placebo [28]; one compared daily norfloxacin with weekly rufloxacin [29]; one cohort study compared fluoroquinolone- or trimethoprim-sulfamethoxazole-based prophylaxis with none in two databases [30]; and one propensity-matched cohort compared trimethoprim-sulfamethoxazole with ciprofloxacin [31].

### 3.2. Risk of Bias

Two of the three pooled trials were at high risk of bias: Elfert 2016 [17] for missing outcome data (recurrence reported for 195 of 262 randomized patients, with differential attrition by arm) and Mostafa 2015 [19] for the randomization process and post-randomization withdrawals. Praharaj 2022 [18] raised some concerns (open label, per-protocol subgroup). Elfert 2016 [17] and Praharaj 2022 [18] were open-label, and Mostafa 2015 [19] was single-blind with the blinded party not specified; none was double-blind. Ginés 1990 [28] and Bauer 2002 [29] raised some concerns. Silvey 2025 [30] was at serious risk (confounding by indication) and Osman 2026 [31] at moderate risk after propensity matching (Supplementary Tables S5 and S6).

### 3.3. Primary Meta-Analysis: Rifaximin Versus Norfloxacin

**Figure 1:**
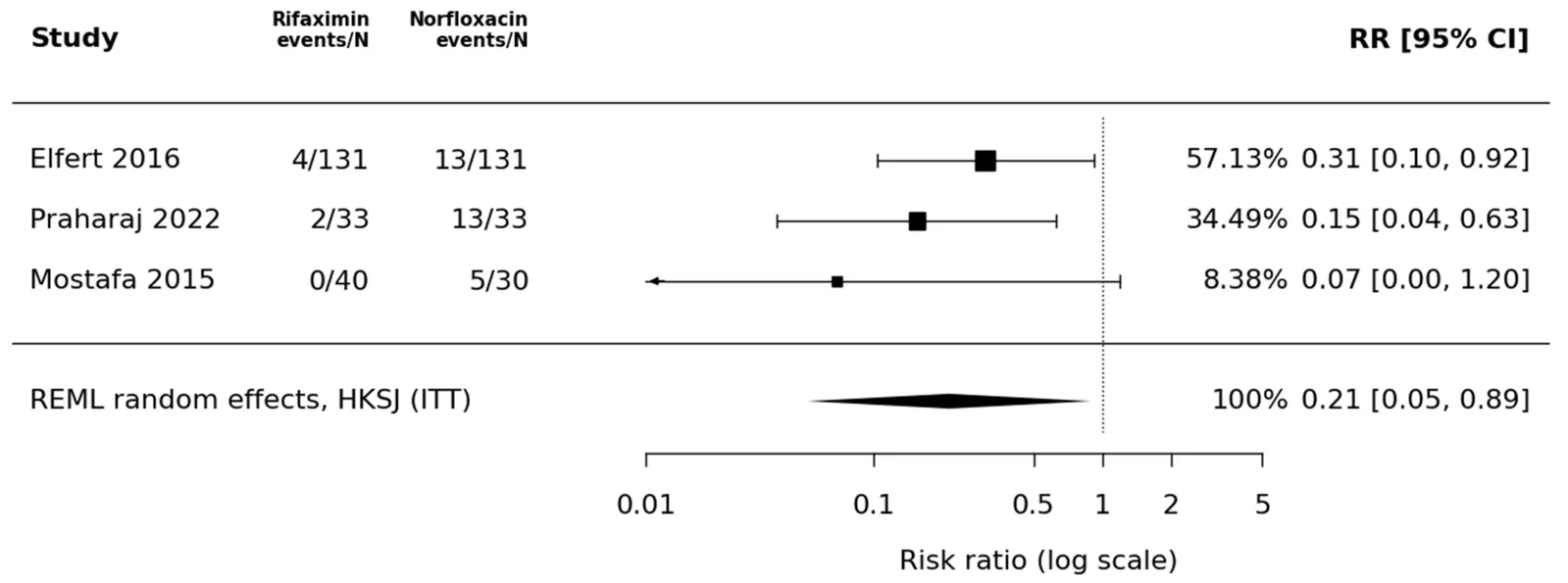
Forest plot of the primary meta-analysis of recurrent spontaneous bacterial peritonitis with rifaximin versus norfloxacin for secondary prophylaxis in patients with cirrhosis (three randomized trials [17,18,19]; intention-to-treat analysis, *n* = 398). Squares represent study-specific risk ratios (RRs), with size proportional to study weight; horizontal lines represent 95% confidence intervals (CIs), and the left-pointing arrow indicates a CI truncated at the axis limit. The diamond represents the pooled estimate from a restricted maximum likelihood (REML) random-effects model with the Hartung–Knapp–Sidik–Jonkman (HKSJ) adjustment: RR 0.21 (95% CI 0.05–0.89; *p* = 0.043; I^2^ = 0%; τ^2^ = 0). A continuity correction of 0.5 was applied to the zero-event trial (Mostafa 2015 [19]) only. Praharaj 2022 [18] denotes the pre-specified rifaximin-versus-norfloxacin subgroup of that trial. Patients with unknown outcomes were counted as recurrence-free, and deaths were treated as competing events (Section 2.5).

Across the three trials, 398 patients were randomized (324 in the trial-reported analysis populations); 6 of 204 rifaximin and 31 of 194 norfloxacin patients had documented recurrence. In the primary intention-to-treat analysis, the pooled RR was 0.21 (95% HKSJ CI 0.05–0.89; *p* = 0.043; I^2^ = 0%, τ^2^ = 0; Figure 1), with study weights of 57.1% (Elfert [17]), 34.5% (Praharaj [18]), and 8.4% (Mostafa [19]). The modified HKSJ interval was 0.03–1.31 (*p* = 0.067). The MH RR without continuity correction was 0.19 (0.08–0.44), and the fixed-study-effects log-link model gave an RR of 0.18 (profile-likelihood 95% CI 0.07–0.39). In the trial-reported populations, the pooled RR was 0.22 (HKSJ 0.07–0.69; modified HKSJ 0.04–1.32; MH 0.19, 0.08–0.45). Because τ^2^ was estimated as zero, no prediction interval distinct from the confidence interval could be computed.

**Clinical heterogeneity.** The three trials differed in rifaximin dose (800, 1100, and 1200 mg/day), population (Mostafa et al. [19] enrolled only Child–Pugh C patients with hepatitis C cirrhosis), outcome ascertainment (protocol paracentesis at 2 and 6 months in Elfert et al. [17] and at 3 and 6 months in Praharaj et al. [18], versus clinical review without protocol paracentesis in Mostafa et al. [19]; Table 1), analysis population (completers, per protocol, and all randomized, respectively), and setting (two trials from Egypt, one from India); all planned six months of treatment and follow-up. These differences could plausibly modify the relative effect, and with three trials the I^2^ statistic has little power to detect them. We therefore do not interpret I^2^ = 0% as evidence of homogeneity; the modified HKSJ interval is reported to guard against an underestimated τ^2^, and inconsistency was considered but not downgraded in the GRADE assessment because all three point estimates favored rifaximin.

### 3.4. Sensitivity Analyses

**Leave-one-out.** In the intention-to-treat analysis, the point estimate remained below unity on omission of each trial (HKSJ RR 0.13, 0.25, and 0.24 omitting Elfert [17], Praharaj [18], and Mostafa [19], respectively), but the HKSJ intervals with one degree of freedom spanned several orders of magnitude and are not interpretable. The MH intervals excluded unity in each case (0.03–0.42, 0.08–0.61, 0.10–0.54). These results show consistency of direction, not robustness of magnitude or significance.

Missing outcome data (Table 2). Outcomes were unknown for 17 surviving non-completers in Elfert 2016 [17] (10 rifaximin, 7 norfloxacin) and for 7 rifaximin patients in Praharaj 2022 [18]; Mostafa 2015 [19] had no missing recurrence data. Assuming recurrence in all of these patients (B1), the pooled RR was 0.65 (HKSJ 0.21–2.03; MH 0.59, 0.37–0.94). Assuming recurrence only among rifaximin patients (B2, worst case for rifaximin), it was 0.80 (HKSJ 0.18–3.44; MH 0.73, 0.45–1.17). Under B3 (best case) and B4 (twice the observed arm-specific rate), the MH RRs were 0.15 (0.07–0.35) and 0.23 (0.11–0.49). The deliberately extreme scenario B5, which imputes recurrence to all 28 rifaximin non-completers in Elfert 2016 [17], including the 18 who died, and to none of the norfloxacin non-completers, gave an MH RR of 1.29 (0.85–1.96) with I^2^ = 88%; because it assumes that every rifaximin death and no norfloxacin death was preceded by recurrence, it does not correspond to the primary estimand and is shown as an extreme bound only. The direction of effect therefore favored rifaximin in every scenario compatible with the primary estimand, but statistical significance was lost when unknown outcomes were assumed to be adverse for rifaximin.

Recurrence or death. In Elfert 2016 [17], the composite was 22 to 32 of 131 with rifaximin and 45 to 52 of 131 with norfloxacin, depending on the 17 patients with unknown status (RR 0.42 to 0.71). In Praharaj 2022 [18], two SBP events and seven deaths occurred among 26 analyzed rifaximin patients and 13 SBP events and 12 deaths among 33 norfloxacin patients, with overlap not reported. Across the resulting bounds, the pooled MH RR for recurrence or death ranged from 0.35 (0.24–0.50) to 0.75 (0.55–1.03), and the REML–HKSJ RR ranged from 0.36 (0.14–0.90) to 0.82 (0.15–4.47); with unknown outcomes assumed event-free, it was 0.41 (0.29–0.58) to 0.46 (0.31–0.67) (Table 2).

Influence. In the trial-reported populations, Elfert 2016 [17] was flagged as influential (Cook’s distance 1.34; hat value 0.57; Supplementary Figure S2).

### 3.5. Secondary Outcomes

All-cause mortality was reported in all three pooled trials and was not pooled. In Elfert 2016 [17], deaths over six months were 18/131 (13.7%) with rifaximin and 32/131 (24.4%) with norfloxacin (RR 0.56, 95% CI 0.33–0.95; randomized population). In the secondary-prophylaxis stratum of Praharaj 2022 [18], 7 of 26 rifaximin and 12 of 33 norfloxacin patients died (26.9% vs. 36.4%; RR 0.74, 0.34–1.61; analyzed population); these counts are stated in the text of that report, whereas the percentages printed in its Table 2 (28.6% vs. 26.9%) do not correspond to them. Mostafa 2015 [19] reported no deaths during six months of follow-up. No trial reported resistant infection as an outcome. Elfert 2016 [17] reported that all culture-positive breakthrough isolates were norfloxacin-resistant (two *Escherichia coli* with rifaximin; three *E. coli*, one *Pseudomonas*, and four Gram-positive cocci with norfloxacin); in Mostafa 2015 [19], all five breakthrough episodes on norfloxacin were caused by Gram-positive cocci; and Praharaj 2022 [18] reported the organisms (*E. coli* and *Stenotrophomonas maltophilia* with norfloxacin) without susceptibility data. Elfert 2016 [17] reported no serious adverse events in either group, and Mostafa 2015 [19] reported no serious adverse reactions, with minor adverse effects (nausea and headache) confined to the norfloxacin arm. Resistant infection and safety therefore could not be synthesized, and this objective is unmet by the available trials.

### 3.6. Narrative Synthesis of Other Comparisons

Prophylaxis versus none. In the 1990 double-blind trial by Ginés et al., norfloxacin reduced the one-year Kaplan–Meier probability of recurrence from 68% to 20% (crude proportions 5/40 vs. 14/40; RR 0.36, 95% CI 0.14–0.89) [28]. In the 2009–2019 VA-CDW (*n* = 4673) and TriNetX (*n* = 6708) cohorts, Silvey et al. found secondary prophylaxis associated with a higher adjusted hazard of recurrence (VA HR 1.63, 95% CI 1.40–1.91; TriNetX HR 1.68) and with fluoroquinolone-resistant organisms in the VA cohort (OR 4.32, 95% CI 1.36–15.83) [30]. Ginés 1990 [28] and Silvey 2025 [30] differ in design, era, comparator, and analysis; they were not pooled, and the contrast between them is descriptive.

*Alternative regimens.* In a propensity-matched TriNetX cohort (2013–2021), trimethoprim-sulfamethoxazole was associated with lower recurrence (HR 0.75, 0.72–0.79) and mortality (HR 0.84, 0.80–0.87) than ciprofloxacin [31]. Bauer et al. found no significant difference between daily norfloxacin and weekly rufloxacin (one-year recurrence 26% vs. 36%) and reported quinolone resistance in all breakthrough isolates [29]. Silvey 2025 [30] (TriNetX) and Osman 2026 [31] overlap in data source and years and were not combined (Supplementary Table S7).

The cohort evidence comprised 11,381 patients in Silvey et al. [30] (VA-CDW 4673; TriNetX 6708) and 22,280 propensity-matched patients in Osman et al. [31] Because both TriNetX analyses draw on the same federated network over overlapping calendar periods (2009–2019 and 2013–2021), some patients probably contribute to both, so the arithmetic total of 33,661 overstates the number of unique patients and is not reported as a combined sample size.

### 3.7. Certainty of Evidence

Certainty for SBP recurrence with rifaximin versus norfloxacin was **very low** (Table 3). Starting from high, we downgraded two levels for risk of bias because the trial contributing 57% of the weight (Elfert 2016 [17]) is at high risk in the domain that drives the result—missing outcome data with differential attrition—and a second trial (Mostafa 2015 [19]) is at high risk for the randomization process, with none double-blind; and one level for imprecision (three trials, 37 events; modified HKSJ interval including no effect; loss of statistical significance under plausible missing-outcome assumptions). We did not downgrade for inconsistency because all three point estimates favored rifaximin, although clinical heterogeneity was considered (Section 3.3), or for indirectness. Publication bias could not be assessed. We considered a rating of low, with a single downgrade for risk of bias, but did not adopt it because the missing-outcome analyses show that the risk-of-bias concern in the weight-dominant trial can remove statistical significance.

## 4. Discussion

### 4.1. Principal Findings

In three randomized trials, none of them double-blind, rifaximin was associated with fewer SBP recurrences than norfloxacin, with a pooled point estimate consistent with previous network meta-analyses [12,13]. What this re-appraisal adds is a characterization of how fragile that estimate is: the interval that guards against underestimated heterogeneity crosses unity; the largest trial reports recurrence for only three-quarters of the patients it randomized, with attrition that was substantial, unequal between arms and driven by death; and a second trial excluded seven randomized patients from one arm only. When death is treated as a competing event, every missing-outcome scenario leaves the point estimate below unity, but assuming that the unknown outcomes were adverse for rifaximin removes statistical significance. The composite of recurrence or death, which does not depend on how deaths are handled, also favors rifaximin across all bounds, with an upper confidence limit reaching 1.03 in the least favorable case. The correct summary is therefore that rifaximin *may* reduce recurrence, with very low certainty—not that its superiority has been demonstrated.

As context rather than as evidence on the primary question, contemporary national cohorts associate fluoroquinolone-based secondary prophylaxis with more, not fewer, recurrences and with fluoroquinolone-resistant infection, in contrast to the placebo-controlled trial on which the practice rests [28,30]. These comparisons (prophylaxis versus none; trimethoprim-sulfamethoxazole versus ciprofloxacin) do not address the rifaximin–norfloxacin question, and the historical–contemporary contrast is confounded by design and era (Section 4.3).

### 4.2. Mechanistic Considerations

Several mechanisms could account for a benefit of rifaximin, if real. Its minimal absorption concentrates the drug in the gut lumen, where bacterial translocation originates [9,10]; it reduces small-intestinal bacterial overgrowth and has been associated with favorable microbiome remodeling rather than the durable dysbiosis produced by systemic fluoroquinolones [11,32]; and it may impose less selective pressure for fluoroquinolone-resistant Enterobacterales, which are over-represented among breakthrough infections on prophylaxis [4,29]. These mechanisms are biologically plausible but were not tested by the included trials, none of which reported microbiological endpoints.

Evidence pointing the other way should be acknowledged. In a prospective cohort of 152 patients, Lutz et al. found no reduction in SBP among patients on rifaximin compared with none, while no patient on systemic prophylaxis developed SBP [33]. That study was predominantly primary prophylaxis, used ciprofloxacin as the comparator, and had a median observation of weeks, so it does not directly contradict the six-month randomized data; but it cautions against assuming that gut-selective activity translates into peritoneal protection.

### 4.3. Interpreting the Historical–Contemporary Contrast

The divergence between Ginés 1990 [28] and Silvey 2025 [30] is striking but has several possible explanations, and our data cannot adjudicate among them. Confounding by indication is the most obvious: patients started on prophylaxis had higher MELD scores and more advanced disease. Differences in comparator (placebo versus no prophylaxis in routine care), outcome ascertainment (trial follow-up versus diagnosis codes), prophylactic agents (norfloxacin versus mixed fluoroquinolones and trimethoprim-sulfamethoxazole), and follow-up all contribute. Rising fluoroquinolone resistance is a plausible additional factor, supported by the calendar-time interaction Silvey et al. [30] observed, the universal quinolone resistance among breakthrough isolates in Bauer et al. [29], the Gram-positive breakthrough infections on norfloxacin in Mostafa et al. [19], and the higher resistance prevalence among patients receiving prophylaxis in related VA analyses [8]. We present this as a hypothesis generated by the contrast, not as a conclusion established by it: era is fully confounded with study design across the available studies, and a meta-regression on calendar year with three data points would be uninformative.

### 4.4. Implications for Practice and Guidelines

Current AASLD, EASL, and APASL guidance positions fluoroquinolones or trimethoprim-sulfamethoxazole as first-line secondary prophylaxis [5,6,34]. Our findings do not justify changing that guidance. They do support three narrower conclusions. First, the randomized evidence that rifaximin is superior to norfloxacin for secondary prophylaxis is of very low certainty and rests on trials whose outcome reporting is incomplete; guideline panels should weight it accordingly. Second, the evidence base for fluoroquinolone secondary prophylaxis itself predates the current resistance landscape, and contemporary observational signals of harm, while confounded, are consistent across two independent databases and warrant formal re-evaluation [8]. Third, the choice between agents should be informed by local fluoroquinolone-resistance prevalence, which the included studies did not stratify.

### 4.5. Future Research

An adequately powered, blinded, contemporary randomized trial of rifaximin versus norfloxacin (and ideally trimethoprim-sulfamethoxazole) for secondary SBP prophylaxis, with intention-to-treat analysis, death handled as a competing event, and microbiological endpoints, remains the clearest need. The difficulty we encountered in reconstructing analysis populations from published reports is itself an argument for participant-level data sharing in prophylaxis trials.

### 4.6. Strengths and Limitations

Strengths include the use of small-sample inference appropriate to three trials, an intention-to-treat estimand with death treated as a competing event, quantitative bounding of the effect under missing-outcome assumptions in every trial with unknown outcomes, a composite outcome that does not depend on how deaths are handled, and verification of all denominators against the participant-flow data of the trial reports.

Limitations are substantial. The review was registered retrospectively in PROSPERO (CRD420261501120) on 8 September 2026, after data extraction and the initial analyses; the restriction of pooling to the rifaximin–norfloxacin comparison was data-informed, and the sensitivity analyses, including the estimand adopted during peer review, were specified after the data were examined. The primary meta-analysis contains three trials, none double-blind, with 37 events, and two trials at high risk of bias; two were conducted in a single country. The pooled estimate is sensitive to unreported outcomes, and trial investigators were not contacted for unreported outcome data, so the missing-outcome and composite analyses rely on published reports only. Rifaximin dose ranged from 800 to 1200 mg/day, outcome ascertainment differed between trials, and one contributing unit was a stratum of a larger trial rather than a standalone trial. Safety and resistant infection could not be synthesized because the trials did not report them systematically. With three trials, I^2^ = 0% provides little assurance of homogeneity, and publication bias cannot be assessed. The observational evidence is subject to confounding by indication and cannot support causal claims about harm from prophylaxis.

## 5. Conclusions

In patients with cirrhosis and prior SBP, rifaximin may reduce recurrence compared with norfloxacin, but the evidence is very uncertain: the estimate is sensitive to unreported outcomes and loses statistical significance under plausible missing-outcome assumptions, although its direction favored rifaximin in every analysis compatible with the primary estimand. This does not justify changing guideline recommendations; it defines the question that a contemporary, blinded, head-to-head trial with intention-to-treat analysis should answer.

## Figures and Tables

**Table 1:** Characteristics of included studies.

Study (Country)	Design	Population	Intervention	Comparator	Analyzed, *n*	SBP Recurrence (Follow-Up); Outcome Ascertainment
Ginés 1990 [28] (Spain)	Double-blind RCT	Cirrhosis, prior SBP	Norfloxacin 400 mg/day	Placebo	40/40	5/40 vs. 14/40 (crude proportions); one-year Kaplan–Meier probability 20% vs. 68% (up to 1 year); ascertainment not extracted (trial not pooled)
Bauer 2002 [29] (Spain)	Open-label multicenter RCT	Cirrhosis, prior SBP	Norfloxacin 400 mg/day	Rufloxacin 400 mg/week	40/39	One-year cumulative recurrence 26% vs. 36% ᵇ (1 year); ascertainment not extracted (trial not pooled)
Mostafa 2015 [19] (Egypt)	Single-blind RCT, 40:30 allocation	Cirrhosis (Child–Pugh C, HCV etiology), prior SBP	Rifaximin 800 mg/day	Norfloxacin 400 mg/day	40/30	0/40 vs. 5/30 (6 months); monthly clinical review, no protocol paracentesis reported
Elfert 2016 [17] (Egypt)	Open-label RCT	Cirrhosis, prior SBP	Rifaximin 1200 mg/day	Norfloxacin 400 mg/day	103/92 ᵃ	4/103 vs. 13/92 (6 months); monthly review, protocol paracentesis at 2 and 6 months
Praharaj 2022 [18] (India)	Open-label RCT, pre-specified secondary-prophylaxis subgroup (NCT03695705)	Cirrhosis, prior SBP	Rifaximin 1100 mg/day (550 mg twice daily)	Norfloxacin 400 mg/day	26/33 (33/33 randomized)	2/26 vs. 13/33 (6 months); monthly review, protocol paracentesis at 3 and 6 months
Silvey 2025 [30] (USA)	Retrospective cohorts (VA-CDW; TriNetX), 2009–2019	Cirrhosis surviving a first SBP episode	Secondary prophylaxis (fluoroquinolone or TMP-SMX)	No prophylaxis	4673; 6708	Adjusted HR 1.63 (95% CI 1.40–1.91, VA-CDW) and 1.68 (TriNetX) (2 years); validated ICD-9/10 codes (index diagnosis confirmed by chart review at two VA centers); recurrence from subsequent coded diagnoses
Osman 2026 [31] (USA)	Propensity-matched retrospective cohort (TriNetX), 2013–2021	Cirrhosis, prior SBP	TMP-SMX	Ciprofloxacin	22,280 (11,140 pairs)	HR 0.75 (95% CI 0.72–0.79) (up to 3 years); ICD-10 code K65.2 in TriNetX structured EHR data; no chart adjudication

Recurrence events are given as rifaximin vs. norfloxacin for the head-to-head trials and as intervention vs. comparator otherwise. CI, confidence interval; EHR, electronic health record; HCV, hepatitis C virus; HR, hazard ratio; ICD, International Classification of Diseases; RCT, randomized controlled trial; SBP, spontaneous bacterial peritonitis; TMP-SMX, trimethoprim–sulfamethoxazole; VA-CDW, Veterans Affairs Corporate Data Warehouse. ᵃ Treatment completers; 131 patients were randomized to each arm. In Praharaj 2022 [18], 33 patients were randomized to each arm of the secondary-prophylaxis stratum. Outcome ascertainment was extracted for the three pooled trials and the two cohort studies; it was not extracted for the two non-pooled trials. ᵇ Event counts not extractable from the published report.

**Table 2:** Missing-outcome and composite-outcome sensitivity analyses (pooled k = 3). Data are events/denominator, rifaximin vs. norfloxacin, for Elfert 2016 [17] and Praharaj 2022 [18]; Mostafa 2015 [19] contributes 0/40 vs. 5/30 to every row (no missing outcomes; no deaths). Unknown outcomes: Elfert, 10 rifaximin and 7 norfloxacin surviving non-completers; Praharaj, 7 rifaximin patients excluded after randomization. Rows C1–C4 bound the number of patients with recurrence or death (Section 2.7). I^2^ values are rounded to the nearest whole percentage point. CI, confidence interval; HKSJ, Hartung–Knapp–Sidik–Jonkman; ITT, intention-to-treat; MH, Mantel–Haenszel; nor, norfloxacin; REML, restricted maximum likelihood; rif, rifaximin; RR, risk ratio.

Scenario	Elfert; Praharaj (Rif vs. Nor)	Pooled RR, REML-HKSJ (95% CI)	Modified HKSJ 95% CI	MH RR (95% CI)	I^2^
Primary: ITT, death as competing event, unknown outcomes event-free	4/131 vs. 13/131; 2/33 vs. 13/33	0.21 (0.05–0.89)	0.03–1.31	0.19 (0.08–0.44)	0%
Trial-reported analysis populations	4/103 vs. 13/92; 2/26 vs. 13/33	0.22 (0.07–0.69)	0.04–1.32	0.19 (0.08–0.45)	0%
B1 all unknown outcomes recurred	14/131 vs. 20/131; 9/33 vs. 13/33	0.65 (0.21–2.03)	0.21–2.03	0.59 (0.37–0.94)	0%
B2 worst case for rifaximin	14/131 vs. 13/131; 9/33 vs. 13/33	0.80 (0.18–3.44)	0.18–3.44	0.73 (0.45–1.17)	0%
B3 best case for rifaximin	4/131 vs. 20/131; 2/33 vs. 13/33	0.17 (0.07–0.41)	0.03–0.99	0.15 (0.07–0.35)	0%
B4 unknown outcomes at 2× observed arm-specific rate	4.8/131 vs. 15.0/131; 3.1/33 vs. 13/33	0.26 (0.08–0.80)	0.05–1.27	0.23 (0.11–0.49)	0%
B5 extreme: recurrence imputed to all rifaximin non-completers in Elfert, including deaths	32/131 vs. 13/131; 9/33 vs. 13/33	0.76 (0.02–37.5)	0.02–37.5	1.29 (0.85–1.96)	88%
C1 recurrence or death, best case for rifaximin	22/131 vs. 52/131; 7/33 vs. 25/33	0.36 (0.14–0.90)	0.14–0.90	0.35 (0.24–0.50)	8%
C2 recurrence or death, unknown event-free; no SBP–death overlap in Praharaj	22/131 vs. 45/131; 9/33 vs. 25/33	0.42 (0.19–0.97)	0.19–0.97	0.41 (0.29–0.58)	0%
C3 recurrence or death, unknown event-free; complete overlap in Praharaj	22/131 vs. 45/131; 7/33 vs. 13/33	0.48 (0.21–1.09)	0.21–1.13	0.46 (0.31–0.67)	0%
C4 recurrence or death, worst case for rifaximin	32/131 vs. 45/131; 16/33 vs. 13/33	0.82 (0.15–4.47)	0.15–4.47	0.75 (0.55–1.03)	51%

**Table 3:** GRADE summary of findings: rifaximin versus norfloxacin for secondary prophylaxis of SBP.

Outcome (Follow-Up)	Assumed Risk, Norfloxacin	Corresponding Risk, Rifaximin (95% CI)	Relative Effect (95% CI)	Participants (Trials)	Certainty	Comments
SBP recurrence (6 months)	167 per 1000	36 per 1000 (9 to 149)	RR 0.21 (0.05 to 0.89); modified HKSJ 0.03 to 1.31	398 (3 RCTs; 324 in trial-reported analyses)	⊕◯◯◯ **Very low** ^a,b^	MH RR 0.15 to 0.73 across plausible missing-outcome scenarios ^c^
SBP recurrence or death (6 months; post hoc)	—	—	MH RR 0.35 to 0.75 across bounds; 0.41 to 0.46 with unknown outcomes event-free	398 (3 RCTs)	⊕◯◯◯ **Very low** ^a,b,d^	Bounds reflect unreported outcomes and unreported SBP–death overlap (Table 2)
All-cause mortality (6 months)	—	—	Not pooled (reported in 3 trials; no deaths in Mostafa 2015 [19])	391 (3 RCTs)	Not rated	Elfert [17]: RR 0.56 (0.33–0.95); Praharaj [18]: 7/26 vs. 12/33, RR 0.74 (0.34–1.61)
Resistant infection/adverse events	—	—	Not reported as an outcome	—	Not rated	Isolate susceptibility reported in Elfert 2016 [17] only (Section 3.5)

^a^ Risk of bias: downgraded two levels. The trial contributing 57% of the weight (Elfert 2016 [17]) is at high risk for missing outcome data with differential attrition, the domain that drives the result, and Mostafa 2015 [19] is at high risk for the randomization process; none of the trials were double-blind. ^b^ Imprecision: downgraded one level. Three trials, 37 events; modified HKSJ interval includes no effect. ^c^ Scenarios B1–B4 (Table 2); a deliberately extreme scenario (B5) gave MH RR 1.29 (0.85–1.96). ^d^ Composite outcome analyzed post hoc; bounds C1–C4 in Table 2. Assumed risk is the median control-arm risk across the three trials. Inconsistency and indirectness: not downgraded. Publication bias: not assessable (k = 3). ⊕◯◯◯ denotes very low certainty of evidence on the four-level GRADE scale. CI, confidence interval; GRADE, Grading of Recommendations Assessment, Development and Evaluation; HKSJ, Hartung–Knapp–Sidik–Jonkman; MH, Mantel–Haenszel; RCT, randomized controlled trial; RR, risk ratio; SBP, spontaneous bacterial peritonitis.

## Data Availability

The original contributions presented in this study are included in the article/Supplementary Materials. All extracted data, the risk-of-bias judgements, and the analysis code are provided in the Supplementary Materials (seven CSV extraction files; Supplementary Code). Further inquiries can be directed to the corresponding author.

## Supplementary Materials

The following supporting information can be downloaded at: https://www.mdpi.com/article/10.3390/biomedicines14102285/s1, Table S1: completed PRISMA 2020 checklist; Table S2: database search strategies and records retrieved; Table S3: reports excluded after full-text assessment, with reasons; Table S4: extracted variables; Table S5: risk of bias of randomized trials (RoB 2); Table S6: risk of bias of cohort studies (ROBINS-I); Table S7: data-source overlap; Figure S1: PRISMA 2020 flow diagram; Figure S2: Baujat plot; Supplementary R code with statistical report, console log and session information; seven Master Extraction CSV files.
