# Supplementary material for: Rifaximin Versus Norfloxacin for Secondary Prophylaxis of Spontaneous Bacterial Peritonitis in the Era of Antimicrobial Resistance: A Systematic Review and Meta-Analysis with Missing-Data Sensitivity Analyses
Source: Biomedicines. 2026 Oct 9;14(10):2285. doi: 10.3390/biomedicines14102285 (PMC13701425; doi:10.3390/biomedicines14102285)

Supplementary Materials

Rifaximin versus Norfloxacin for Secondary Prophylaxis of Spontaneous Bacterial Peritonitis in the Era of Antimicrobial Resistance

Supplementary Code: Reproducible R analysis

The reproducibility package Supplementary_R_code_bundle_20260926.zip contains Rrevisionanalyses_corrected.r, the complete primary-run output Rrevisionanalyses_corrected_output.txt, the second-run console record R_warnings.txt, and Supplementary_R_code_README.md. The corrected script reproduces the primary intention-to-treat analysis with death as a competing event, the trial-reported analysis-population sensitivity analysis, the modified HKSJ interval, Mantel–Haenszel RR without continuity correction, the fixed-study-effects log-link RR model, leave-one-out analyses, post hoc missing-outcome scenarios B1–B5, and the recurrence-or-death composite bounds C1–C4. The validated run used R 4.3.3 and metafor 4.4-0.

Validated headline results: primary ITT REML–HKSJ RR 0.214 (95% CI 0.051–0.893), reported in the manuscript as RR 0.21 (0.05–0.89); modified HKSJ interval 0.0348–1.313, reported as 0.03–1.31; MH RR without continuity correction 0.189 (0.082–0.436), reported as 0.19 (0.08–0.44); and fixed-study-effects log-link RR 0.1838 (profile-likelihood 95% CI 0.0708–0.3948), reported as 0.18 (0.07–0.39). Study weights were 57.13% for Elfert 2016, 34.49% for Praharaj 2022, and 8.38% for Mostafa 2015. The trial-reported analysis-population sensitivity estimate was RR 0.22 (HKSJ 0.07–0.69; modified HKSJ 0.04–1.32; MH 0.19, 0.08–0.45).

Supplementary Data: Master Extraction Sheet

The master extraction data are provided as seven CSV files. The filenames below are the exact files submitted with this article:

- **Main Extraction** (Master_Extraction_Sheet_v3_3_Main_Extraction_v3.csv) contains the full data extraction.
- **For Meta-Analysis** (Master_Extraction_Sheet_v3_3_For_Meta_Analysis.csv) contains the 2 × 2 data used for pooling.
- **Risk of Bias Judgements** (Master_Extraction_Sheet_v3_3_RoB_2_RCTs.csv and Master_Extraction_Sheet_v3_3_ROBINS_I_Cohorts.csv).
- **Overlap Matrix and Pairwise Summary** (Master_Extraction_Sheet_v3_3_Overlap_Matrix_v3.csv and Master_Extraction_Sheet_v3_3_Pairwise_Summary_v3.csv).

- **Audit Log (Master_Extraction_Sheet_v3_3_Audit_Log_v3_2.csv) records all corrections and analytic decisions made during extraction and peer review, including the intention-to-treat structure (398 randomized patients), the 33-row scenario matrix used for the primary, trial-reported, B1–B5 and C1–C4 analyses, and verification of study-level descriptive data against the source reports. The seven CSV files and the R code constitute the data and analysis record for this review.**

Statistical analysis summary

The primary estimand is the intention-to-treat RR for six-month SBP recurrence across all 398 randomized patients, with death treated as a competing event and non-death unknown outcomes initially treated as recurrence-free. The trial-reported analysis populations (324 of 398 randomized patients) are retained as a sensitivity analysis. The three pooled trials contributed 6/204 recurrence events in the rifaximin arm and 31/194 in the norfloxacin arm.

Post hoc missing-outcome scenarios are labeled B1–B5. B1 assumes all unknown outcomes recur; B2 imputes recurrence only to rifaximin patients; B3 imputes recurrence only to norfloxacin patients; B4 applies twice the observed arm-specific risk; and B5 is the deliberately extreme comparison scenario that also imputes recurrence to rifaximin deaths. The recurrence-or-death composite is reported in bounds C1–C4. The primary ITT MH RR is 0.19 (0.08–0.44); plausible B1–B4 MH RRs range from 0.15 to 0.73, while the extreme B5 MH RR is 1.29 (0.85–1.96). Composite MH RRs range from 0.35 (0.24–0.50) to 0.75 (0.55–1.03).

The seven CSV files are the data and audit layer for this analysis: Main Extraction, For Meta-Analysis, RoB 2 RCTs, ROBINS-I Cohorts, Overlap Matrix, Pairwise Summary, and Audit Log. The manuscript reports very low GRADE certainty for the primary recurrence outcome and the post hoc recurrence-or-death composite; the completed checklist and risk-of-bias tables below use the section and table numbering of the article.

Table S1. PRISMA 2020 Checklist

Manuscript: Rifaximin versus Norfloxacin for Secondary Prophylaxis of Spontaneous Bacterial Peritonitis in the Era of Antimicrobial Resistance

Item	Topic	Location reported
1	Title	Title page
2	Abstract	Structured Abstract
3	Rationale	Introduction, paragraphs 1–4
4	Objectives	Introduction, final paragraph
5	Eligibility criteria	Methods, Section 2.3
6	Information sources	Methods, Section 2.2; Supplementary Table S2
7	Search strategy	Supplementary Table S2
8	Selection process	Methods, Sections 2.2–2.4
9	Data collection process	Methods, Section 2.4
10a	Outcome data items	Methods, Sections 2.4–2.5; Supplementary Table S4
10b	Other data items	Methods, Section 2.4; Supplementary Table S4
11	Risk-of-bias assessment	Methods, Section 2.6; Supplementary Tables S5–S6
12	Effect measures	Methods, Sections 2.5 and 2.7
13a	Eligibility for each synthesis	Methods, Section 2.7
13b	Data preparation	Methods, Sections 2.4 and 2.7
13c	Tabulation and visual display	Table 1; Figures 1, S1 and S2

Item	Topic	Location reported
13d	Synthesis methods	Methods, Section 2.7
13e	Exploration of heterogeneity	Methods, Section 2.7; Results, Sections 3.3–3.4
13f	Sensitivity analyses	Methods, Section 2.7; Results, Section 3.4; Table 2
14	Reporting-bias assessment	Methods, Section 2.7
15	Certainty assessment	Methods, Section 2.6
16a	Study selection results	Results, Section 3.1; Figure S1
16b	Excluded studies	Supplementary Table S3
17	Study characteristics	Results, Section 3.1; Table 1
18	Risk-of-bias results	Results, Section 3.2; Supplementary Tables S5–S6
19	Individual-study results	Table 1; Figure 1
20a	Contributing studies	Results, Section 3.3; Figure 1
20b	Statistical synthesis results	Results, Section 3.3; Figure 1
20c	Heterogeneity	Results, Sections 3.3–3.4
20d	Sensitivity analyses	Results, Section 3.4; Table 2; Figure S2
21	Reporting biases	Results, Section 3.7
22	Certainty of evidence	Results, Section 3.7; Table 3
23a	Interpretation	Discussion, Sections 4.1–4.4
23b	Limitations of evidence	Discussion, Section 4.6
23c	Limitations of review process	Discussion, Section 4.6
23d	Implications	Discussion, Sections 4.4–4.5
24a	Registration	Methods, Section 2.1: PROSPERO CRD420261501120 (registered retrospectively on 8 September 2026)
24b	Protocol	Methods, Section 2.1: no public protocol
24c	Amendments	Methods, Section 2.1
25	Support	Funding statement
26	Competing interests	Conflicts of Interest statement
27	Data/code/materials availability	Data Availability Statement; supplementary files

Table S2. Database search strategies and records retrieved

Database	Last search date	Search strategy	Records retrieved
MEDLINE via PubMed	15 July 2026	("spontaneous bacterial peritonitis" OR SBP) AND (prophylaxis OR prevention) AND (cirrhosis OR "liver cirrhosis") AND (rifaximin OR norfloxacin OR ciprofloxacin OR trimethoprim-sulfamethoxazole)	103
Embase	15 July 2026	('spontaneous bacterial peritonitis'/exp OR ('spontaneous bacterial peritonitis' OR SBP):ti,ab,kw) AND ('liver cirrhosis'/exp OR (cirrhosis OR 'liver cirrhosis' OR 'hepatic cirrhosis'):ti,ab,kw) AND (prophylax* OR prevent* OR recurrence OR recurrent):ti,ab,kw AND ('rifaximin'/exp OR rifaximin:ti,ab,kw OR Xifaxan:ti,ab,kw OR 'norfloxacin'/exp OR norfloxacin:ti,ab,kw OR 'ciprofloxacin'/exp OR ciprofloxacin:ti,ab,kw OR 'co-trimoxazole'/exp OR ('trimethoprim-sulfamethoxazole' OR 'trimethoprim sulfamethoxazole' OR cotrimoxazole OR 'co-trimoxazole' OR TMP-SMX):ti,ab,kw)	126
CENTRAL	15 July 2026	("spontaneous bacterial peritonitis" OR SBP) AND (prophylaxis OR prevention) AND cirrhosis AND (rifaximin OR norfloxacin OR ciprofloxacin OR trimethoprim-sulfamethoxazole)	68
Scopus	15 July 2026	TITLE-ABS-KEY("spontaneous bacterial peritonitis" OR SBP) AND TITLE-ABS-KEY(prophylaxis OR prevention) AND TITLE-ABS-KEY(cirrhosis) AND TITLE-ABS-KEY(rifaximin OR norfloxacin OR ciprofloxacin OR "trimethoprim-sulfamethoxazole")	149

Table S3. Reports excluded after full-text assessment, with reasons

Citation	Reason for exclusion
Kuo CH et al. 2025	Rifaximin and antimicrobial resistance in cirrhosis/hepatic encephalopathy; not a secondary-SBP-prophylaxis head-to-head comparison with norfloxacin
Bajaj JS et al. 2026	Pre-admission rifaximin and drug-resistant infections; wrong exposure/comparison for the focused review
Merli M et al. 2015	MDR/XDR infection survey; no defined rifaximin-norfloxacin secondary-prophylaxis comparison
Hillert A et al. 2021	Resistance profile in infected decompensated cirrhosis; no defined prophylaxis comparison
Dalbeni A et al. 2023	MDRO and antimicrobial-stewardship cohort; no defined rifaximin-norfloxacin comparison
Alexopoulou A et al. 2016	Prognostic study of XDR infection and mortality; no prophylaxis comparison
Kimmann M et al. 2019	Nosocomial SBP outcomes by disease stage; no prophylaxis comparison
Trad N et al. 2024	Clinical impact of MDRO infection; no defined prophylaxis comparison
Silvey S et al. 2025 CTG	Non-SBP infection endpoint; outside the prespecified focused rifaximin-norfloxacin secondary-prophylaxis comparison
Goel A et al. 2017	Systematic review/meta-analysis rather than an eligible primary RCT or cohort report

Table S4. Extracted variables

Study identifiers (first author, year, country, registration); design; eligibility and prior-SBP definition; randomized, analyzed and excluded sample sizes per arm; age, sex, Child–Pugh class and MELD; intervention and comparator (agent, dose, frequency and duration); follow-up; SBP recurrence (events and denominator with analysis population stated); outcome ascertainment; all-cause mortality and whether death is a competing event; unknown outcomes and participant-flow reasons; resistant or MDR infection; adverse events; funding; and data-source overlap.

Table S4 (continued). Study-level descriptive data

Silvey 2025: VA adjusted liver-transplant OR 1.85 (95% CI 1.20–2.94); TriNetX alcohol etiology 53.2% overall (50.4% without prophylaxis and 56.1% with prophylaxis). These cohorts remain narrative context and are not pooled with the RCT estimand.

Elfert 2016: enrollment 2014; age 53.78 vs 54.12 years; male sex 56.5% vs 51.9%; etiology not reported; mortality 18/131 vs 32/131, RR 0.563 (95% CI 0.33–0.95). Recurrence is 4/131 vs 13/131 in primary ITT and 4/103 vs 13/92 in the trial-reported completer analysis.

Mostafa 2015: enrollment May 2013–March 2014; age 55.8 vs 56.5 years; etiology 100% HCV; recurrence 0/40 vs 5/30; no deaths occurred in either arm; single-blind with the blinded party not specified.

Praharaj 2022: DOI 10.1016/j.jceh.2021.08.010; PGIMER Chandigarh; enrollment in 2016; age 47.5 vs 45.8 years; male sex 61.5% vs 75.8%; subgroup etiology not reported.

Ginés 1990: multicenter Spain; study period NR; mean follow-up 6.4 ± 0.6 months; one-year Kaplan–Meier outcome wording; age, sex and etiology not extracted.

Osman 2026: after matching, age 61.2 vs 61.3 years and male sex 59.5% vs 59.2% for TMP-SMX vs ciprofloxacin; outcomes assessed from 1 through 1095 days. The recurrence HR was 0.75 (0.72–0.79) and mortality HR was 0.84 (0.80–0.87).

Table S5. Risk of bias (RoB 2) — randomized trials

Study	D1 Randomization	D2 Deviations	D3 Missing data	D4 Measurement	D5 Selection	Overall
Ginés 1990	Some concerns	Low	Some concerns	Low	Some concerns	**Some concerns**
Bauer 2002	Some concerns	Some concerns	Some concerns	Low	Some concerns	**Some concerns**
Mostafa 2015	High	High	High	Low	Some concerns	**High**
Elfert 2016	Low	Some concerns	High	Low	Low	**High**
Praharaj 2022	Low	Some concerns	Some concerns	Low	Low	**Some concerns**

Justifications. Ginés 1990: multicenter double-blind placebo-controlled trial; sequence generation and concealment were not fully described, so the unverified domains remain some concerns. Bauer 2002: open-label; concealment and participant flow were

incompletely reported. Mostafa 2015: unequal allocation (40:30) with unclear method; single-blind, with the blinded party not specified; five norfloxacin patients were withdrawn after recurrence, with post-randomization handling unclear. Elfert 2016: computer-generated sequence, permuted blocks of eight and sequentially numbered opaque sealed envelopes (D1 low); open-label (D2); recurrence reported for completers only (103/131 and 92/131), with differential attrition driven largely by death (D3 high). Praharaj 2022: sealed envelopes and pre-registered (NCT03695705); open-label; secondary-prophylaxis stratum randomized 33 patients per arm, with 7 rifaximin patients excluded post-randomization and 26/33 vs 33/33 analyzed per protocol.

Table S6. Risk of bias (ROBINS-I) — cohort studies

Study	Confounding	Selection	Classification	Deviations	Missing	Measurement	Reporting	Overall
Silvey 2025 (VA-CDW)	Serious	Moderate	Moderate	Low	Low	Low	Low	**Serious**
Silvey 2025 (TriNetX)	Serious	Moderate	Serious	Low	Moderate	Low	Low	**Serious**
Osman 2026	Moderate	Low	Low	Low	Low	Low	Low	**Moderate**

Justifications. Silvey 2025: confounding by indication was addressed by multivariable adjustment only; VA-CDW index SBP was confirmed by chart review at two VA centers and recurrence was identified from subsequent coded diagnoses; TriNetX exposure was defined by a single prescription. Osman 2026: structured TriNetX ICD-10 code K65.2 outcomes without chart adjudication; 1:1 propensity matching (n = 22,280); post-match age 61.2 vs 61.3 years and male sex 59.5% vs 59.2%; outcomes assessed up to 1095 days; residual unmeasured confounding remains possible.

Table S7. Data-source overlap

Source	Studies	Overlap	Handling
VA-CDW	Silvey 2025	—	Single study
TriNetX	Silvey 2025; Osman 2026	Moderate (overlapping years, different comparisons)	Not combined
Egypt (different centers)	Mostafa 2015; Elfert 2016	Low	Different hospitals (Tanta University; National Liver Institute, Menoufiya); enrollment periods partly overlap (2014; May 2013–March 2014); no indication of shared patients; both pooled
India (PGIMER Chandigarh)	Praharaj 2022	—	Single study
Spain (multicenter)	Ginés 1990; Bauer 2002	Low	Not pooled; enrollment periods not verified

Figure S1. PRISMA 2020 flow diagram

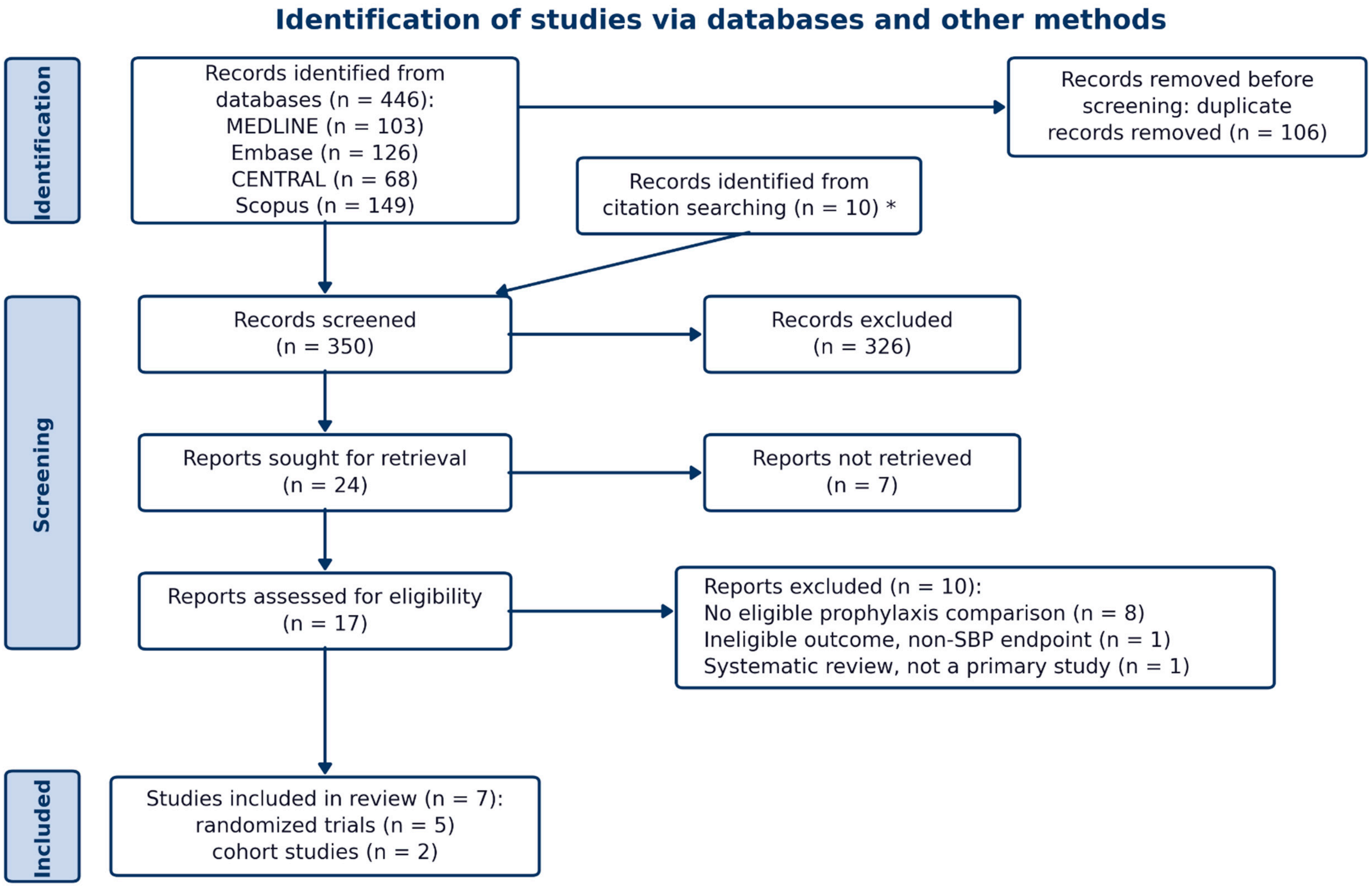

* Records identified through citation searching (reference lists of prior systematic reviews) were pooled with the database records before deduplication; counts from screening onward therefore refer to the combined set of records.

PRISMA 2020 flow diagram. From: Page MJ, et al. BMJ 2021;372:n71.

Figure S2. Baujat plot (k = 3)

Contribution of each trial to heterogeneity (x-axis) against influence on the pooled estimate (y-axis), based on the trial-reported analysis populations. Elfert 2016 exerts the greatest influence (Cook's distance 1.34; hat value 0.57).

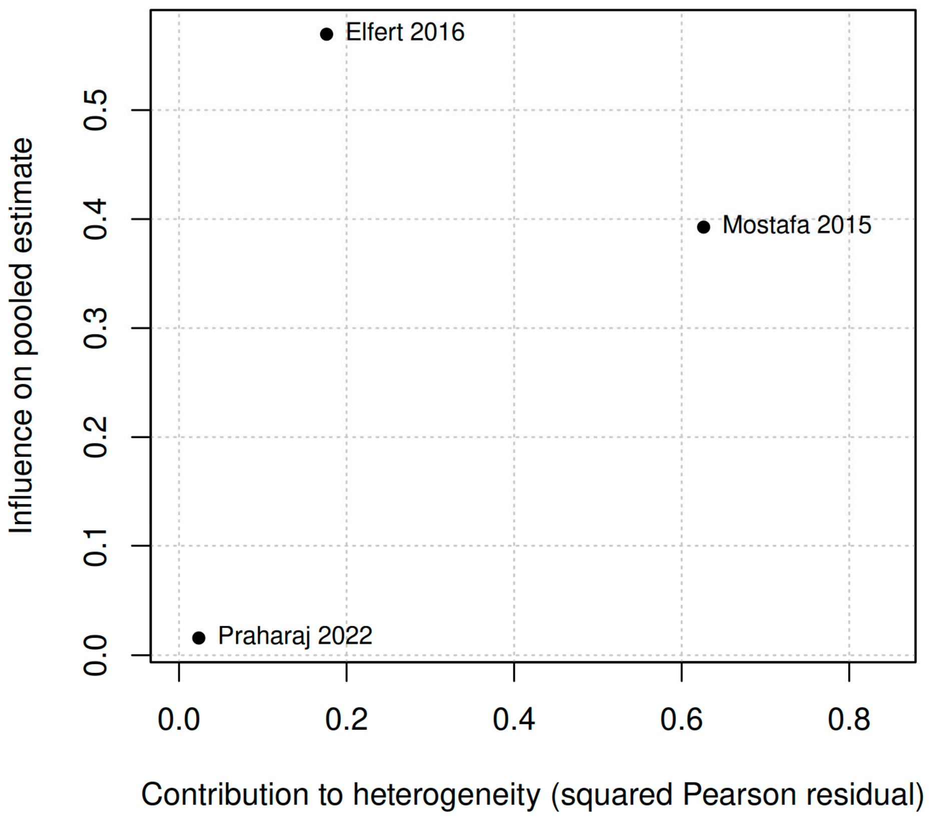

Supplement: Supplementary file 1 [file biomedicines-14-02285-s001.zip › biomedicines-4599652_Supplementary_Materials_proof_clean.pdf]
